# Temporal Variability of ECG Risk Markers and Clinical Outcomes in Non-Dilated Left Ventricular Cardiomyopathy

**DOI:** 10.3390/jcm15020402

**Published:** 2026-01-06

**Authors:** Nikias Milaras, Konstantinos Pamporis, Konstantinos A. Gatzoulis, Paschalis Karakasis, Panagiotis Kostakis, Zoi Sotiriou, Anastasia Xintarakou, Ageliki Laina, Dimitrios Karelas, Dimitrios Vlachomitros, Iosif Xenogiannis, Stefanos Archontakis, Charalampos Vlachopoulos, Konstantinos Toutouzas, Konstantinos Tsioufis, Skevos Sideris

**Affiliations:** 1 School of Medicine, National and Kapodistrian University of Athens, “Hippokration” General Hospital of Athens, 11527 Athens, Greece; konstantinospab@gmail.com (K.P.); kgatzoul@med.uoa.gr (K.A.G.); dim.f.karelas@gmail.com (D.K.);; 2 State Department of Cardiology, “Hippokration” General Hospital of Athens, 11527 Athens, Greece; panos_kost@hotmail.com (P.K.);; 3 Second Department of Cardiology, Aristotle University of Thessaloniki, “Hippokration” General Hospital of Thessaloniki, 54642 Thessaloniki, Greece; pakar15@hotmail.com; 4 First University Cardiology Department, School of Medicine, National and Kapodistrian University of Athens, “Hippokration” General Hospital of Athens, 11527 Athens, Greece

**Keywords:** non-dilated left ventricular cardiomyopathy, ventricular tachycardia, VF, Holter, SAECG

## Abstract

**Background/Objectives**: Non-dilated left ventricular cardiomyopathy (NDLVC) is a recently defined clinical entity associated with increased risk of ventricular arrhythmias (VA) and sudden cardiac death (SCD), despite preserved LV geometry. The role and temporal variability of noninvasive electrocardiographic (ECG) risk markers in this population remain insufficiently characterized. To assess the temporal variability of ECG-derived risk markers in patients with NDLVC and explore their association with major adverse cardiac events, including heart failure (HF) and VA hospitalization. **Methods**: We prospectively studied 55 patients with NDLVC who underwent cardiac magnetic resonance imaging and serial 24 h Holter monitoring, signal-averaged ECG, and standard 12-lead ECG over a one-year period. Patients were followed up for 39.5 ± 8.6 months. Nine ECG-based risk markers were analyzed, including premature ventricular contraction (PVC) burden, non-sustained ventricular tachycardia (NSVT) occurrence, its maximum rate and maximum beats, mean QTc interval, standard deviation of NN intervals (SDNN), deceleration capacity (DC), heart rate turbulence onset and slope (TO/TS), T-wave alternans (TWA), and late potentials. Clinical outcomes were HF and VA hospitalization. Logistic regression was used to evaluate associations between changes in ECG parameters and outcomes. **Results**: A change (from positive to negative and vice versa) in at least one ECG parameter was detected in 67.3% of patients, with the highest variability observed in TWA (34.5%), NSVT (23.6%), and PVC burden (23.6%). Despite this variability, only SDNN was significantly associated with increased risk of VA hospitalization during follow-up (OR = 0.98, 95% CI: 0.97–0.99, *p* = 0.006). No ECG changes were associated with HF hospitalization. **Conclusions**: Patients with NDLVC exhibit substantial temporal variability in noninvasive ECG risk markers. While most changes do not correlate with clinical events, an inverse association was found between SDNN and VA risk. These findings support the ongoing evaluation and the necessity to identify more effective risk stratification markers in this subgroup of patients.

## 1. Introduction

The role of electrocardiographic (ECG) noninvasive risk factors in assessing the risk of sudden cardiac death (SCD) in patients with non-dilated left ventricular cardiomyopathy (NDLVC) remains insufficiently explored, as is their temporal variability.

NDLVC is a clinical entity that has only recently been formally defined and incorporated into clinical practice guidelines [1]. As such, data regarding its natural history, prognostic implications, and optimal management strategies remain limited. Most of the existing literature on non-ischemic cardiomyopathies has historically centered on patients with overt ventricular dilatation, particularly those meeting diagnostic criteria for classic dilated cardiomyopathy (DCM). In contrast, individuals with preserved left ventricular (LV) geometry but impaired function were often underrepresented or variably classified across studies. NDLVC represents a subset of patients who were previously grouped under the broader DCM spectrum. It encompasses a heterogeneous group of etiologies and exhibits a range of structural and functional cardiac phenotypes. According to the 2023 consensus nomenclature, NDLVC is defined by the presence of non-ischemic left ventricular fibrosis or fatty infiltration, regardless of the presence of global or regional wall motion abnormalities. It also includes patients with isolated global hypokinesia without detectable scar, or with myocardial fibrosis evident on imaging, even in the context of normal LV dimensions [1]. The NDLVC spectrum may therefore encompass individuals previously diagnosed with non-dilated forms of DCM, or arrhythmogenic cardiomyopathy phenotypes.

Despite its generally more preserved LV geometry, the clinical course of NDLVC remains poorly defined. Importantly, patients with NDLVC appear to be at increased risk for life-threatening ventricular arrhythmias (VA) and SCD, while current clinical guidelines do not adequately address this population [2]. Recommendations for implantable cardioverter–defibrillator (ICD) implantation continue to rely primarily on LVEF thresholds derived from studies in classic DCM. This poses a significant limitation, as many patients with NDLVC demonstrate preserved or only mildly reduced systolic function, and may not meet conventional criteria for primary prevention ICD therapy despite being at substantial arrhythmic risk [3]. Therefore, there is an urgent need for more refined risk stratification criteria that go beyond LVEF, incorporating ECG, imaging, and genetic or biomarker-based parameters to guide individualized management.

This study aimed to document the baseline clinical, ECG, and imaging characteristics of patients with NDLVC, and determine whether close follow-up using serial 24 h Holter monitoring, standard ECG, and signal-averaged ECG (SAECG) improves the identification of patients who are at risk for HF and VA hospitalization.

## 2. Methods

### 2.1. Study Population

This was a prospective observational cohort study. Reporting of the presented study was performed based on the Strengthening the Reporting of Observational Studies in Epidemiology (STROBE) guidelines [4]. Between January 2020 and June 2025, patients diagnosed with non-dilated left ventricular cardiomyopathy (NDLVC) were consecutively enrolled. Inclusion criteria evolved over the study period: from 2020 to 2023, eligibility was based on the 2016 definition of hypokinetic non-dilated cardiomyopathy [3], whereas from 2023 onward, the updated NDLVC criteria were adopted [1]. Importantly, all included patients fulfilled the current diagnostic criteria for NDLVC across the study cohort. Participants were recruited at a single tertiary care center—the General Hospital of Athens Ippokrateion—during routine outpatient evaluations at either the Heart Failure or Arrhythmia Clinics. Eligibility was assessed based on predefined clinical, imaging, and electrophysiological criteria at the time of initial presentation, as detailed in subsequent sections. The study was conducted in accordance with the ethical principles of the Declaration of Helsinki and received approval from the Institutional Review Board of the institution (Approval No. 22843/30 December 2022). Written informed consent was obtained from all participants prior to enrollment.

### 2.2. Eligibility Criteria

#### 2.2.1. Inclusion Criteria

- Adult patients aged 18–80 years.
- NDLVC was defined as the absence of LV dilatation on CMR (indexed for age, sex, and body surface area) in combination with either (a) non-ischemic LV myocardial fibrosis detected by late gadolinium enhancement or (b) reduced LVEF < 50% in the absence of detectable myocardial scar [5].
- Obstructive coronary artery disease had to be ruled out through coronary angiography.
- All patients should have been diagnosed as having cardiomyopathy and receive guideline-directed medical therapy for at least 6 months before inclusion, in order to exclude reversible causes.
- Furthermore, patients were required to be in sinus rhythm or paroxysmal atrial fibrillation and have <10% burden of premature ventricular complexes (PVCs)/24 h in Holter monitoring in order to exclude possible arrhythmia-induced cardiomyopathy.

#### 2.2.2. Exclusion Criteria

- Use of any antiarrhythmic agents besides beta blockers.
- Indication for permanent pacing, either for bradyarrhythmia prevention or cardiac resynchronization therapy.

### 2.3. Clinical, Imaging, and Electrocardiographic Data Acquisition

All participants underwent comprehensive clinical evaluation, including collection of somatometric, demographic, and pharmacological data. CMR with gadolinium-based contrast enhancement was performed in all patients to assess structural and functional parameters. Specifically, left and right ventricular end-systolic and end-diastolic volumes, ejection fraction of both ventricles, and the presence and distribution of late gadolinium enhancement (LGE) were measured. CMR studies were conducted using either 1.5 T or 3.0 T scanners equipped with cardiac phased-array surface coils, ECG gating, and breath-hold protocols, utilizing dedicated cardiac imaging software.

To assess the electrophysiological substrate, all patients underwent 24 h Holter monitoring (CardioMem^®^ CM 4000, GETEMED, Teltow, Germany), resting 12-lead ECG, and signal-averaged ECG (SAECG) using the MAC 5500 HD system (GE Healthcare, Chicago, IL, USA). All Holter recordings were performed using the same recording system. The device was applied in the morning hours and recordings were standardized to a continuous 24 h duration. Patients were instructed to maintain their usual daily activities.

The same ECG modalities were re-evaluated in each patient in the first yearly follow-up visit. Seven electrocardiographic parameters were systematically evaluated:

**(a)**  QRS Duration and Morphology on surface ECG. Patients with QRS duration > 120 ms were further categorized as having left bundle branch block (LBBB), right bundle branch block (RBBB), or non-specific intraventricular conduction delay (NS-IVCD).
**(b)**  Late Potentials (LPs) on SAECG over a 45 min resting period. LPs were considered present if ≥2 of the following criteria were met [6]: filtered QRS duration > 114 ms; low-amplitude (<40 μV) signal duration > 38 ms in the terminal portion of the QRS complex; and root mean square (RMS) voltage of the final 40 ms < 20 μV. For patients with QRS duration > 120 ms, modified diagnostic criteria were applied as per Gatzoulis et al. [7].
**(c)**  Premature Ventricular Contractions (PVCs), total number recorded over 24 h Holter monitoring [6]. This index was considered as a high-risk marker when PVCs > 1000/24 h were recorded [8].
**(d)**  Non-Sustained Ventricular Tachycardia (NSVT) occurrence, the number of maximum beats per NSVT run and its fastest rate, also derived from 24 h Holter monitoring. NSVT was defined as ≥3 consecutive premature ventricular beats at a rate > 100 bpm and lasting <30 s [9,10].
**(e)**  Standard Deviation of NN Intervals (SDNN) ≤ 75 ms. This heart rate variability index was calculated from 24 h Holter data, with lower SDNN values reflecting impaired autonomic regulation and increased cardiovascular risk [11].
**(f)**  Heart rate turbulence which was measured in a 24 h Holter recording with the assumption of the existence of a sufficient number of PVCs. It is quantified by turbulence onset (TO) and turbulence slope (TS) which represent the initial acceleration and subsequent deceleration of the sinus after a PVC. TO was considered pathological when ≥0% and TS when ≤2.5 ms/R-R interval [12,13]. Additionally, we analyzed the combined presence of TO ≥ 0% and TS ≤ 2.5 ms/R-R interval, as suggested by previous studies [13]. Heart rate turbulence parameters were calculated only in patients with ≥5 suitable isolated PVCs during Holter monitoring, in accordance with established methodological standards.
**(g)**  Deceleration capacity (DC) is an index that quantifies the deceleration of the heart rate from 24 h ECG monitoring. It is described as a metric that provides a specific and independent assessment of parasympathetic tone within the autonomic nervous system [14,15]. DC below 4.5 ms was considered abnormal.
**(h)**  Fridericia-Corrected mean QT Interval (QTc) derived from 24 h Holter recordings. QTc prolongation was defined as >440 ms in men and >450 ms in women [16].
**(i)**  Ambulatory T-Wave Alternans (TWA) ≥ 65 μV, detected in at least two Holter leads using the modified moving average method. TWA refers to beat-to-beat alternation in T-wave amplitude or morphology and is considered a marker of electrical instability and elevated arrhythmic risk [17].

### 2.4. Study Endpoints

The endpoints of the present study were as follows:

- The differences in the studied ECG risk markers as derived through 24 h Holter monitoring between two visits that were scheduled a year apart;
- HF hospitalization, which was defined as admission for ≥24 h with a primary diagnosis of heart failure, with ≥1 symptom and ≥2 physical examinations, laboratory, or invasive findings of heart failure, and the patient receives heart-failure-specific treatment [18];
- Hospitalization for VA, including VT/VF occurrence or SCD. SCD was defined as unexpected death due to cardiac causes with or without documented VA, death within 1 h of acute symptoms, or nocturnal death with no antecedent history of immediate worsening symptoms [19]. VT/VF occurrence included sustained (>30 s) VT causing hemodynamic instability or appropriate implantable cardioverter–defibrillator (ICD) interventions, defined as a shock for termination of rapid (>173 beats/min) sustained VT or VF.

All patients underwent systematic phenotyping and follow-up. In-person follow-up visits were scheduled for all participants at 12 months and then annually following study inclusion. During the first visit, patients were scheduled to undergo CMR, 24 h Holter monitoring, SAECG, resting ECG, and echocardiography. In their second visit after one year, patients did not have to undergo a repeat CMR while they were re-evaluated with every other aforementioned modality. After the first two visits, patients were then re-examined annually. In addition, participants were instructed to promptly notify the study team of any interim hospitalizations to ensure accurate event capture.

### 2.5. Statistical Analysis

Categorical variables are presented as frequencies with percentages, while continuous variables as mean and standard deviation (SD) when normally distributed, or as median with interquartile range (IQR) when non-normally distributed. Normality of variables was assessed with QQ-plots and formally tested with the Shapiro–Wilk test. Missing data were only encountered in 9/55 (16%) of the TO and TS variables due to calculation problems of the Holter, with no missing data occurring the rest of the dataset. To estimate differences in Holter parameters between the examined time points, the paired *t*-test (using the Welch’s correction as appropriate) was used for normally distributed variables and the Wilcoxon signed-rank test for non-normally distributed variables. Respectively, to examine differences in categorical variables, the McNemar’s test was used to account for paired observations. Furthermore, to estimate the percentage of change in time across the levels of categorical Holter parameters, a one-sample z test for proportions was used to calculate point estimates with 95% confidence intervals (CI). To assess temporal variability in arrhythmic substrate and autonomic function markers, we evaluated the proportion of patients who shifted from a positive to a negative risk marker (or vice versa) between visits. Finally, to explore potential associations between changes in Holter parameters and clinical outcomes (HF and VA hospitalization), univariate logistic regression models were used with calculation of odds ratio (OR) with 95% CI. All models were assessed for validity of logistic regression assumptions. A two-sided *p*-value < 0.05 was considered significant for all analyses. All analyses were performed with R statistical software (v. 4.4.2). To further assess whether the presence of LGE modified the association between SDNN, TS, and VA hospitalization, an interaction term was added to the univariate models. To explore whether a clinically meaningful threshold of SDNN and TS could improve discrimination of VA-related hospitalization, receiver operating characteristic (ROC) analysis was performed. The optimal cutoff value was defined as the point maximizing the Youden index (sensitivity + specificity − 1).

## 3. Results

### 3.1. Baseline Characteristics

A total of seventy-one patients were initially considered for inclusion in this study. Nine were excluded due to inability to undergo CMR imaging and seven patients did not have follow-up Holter monitoring data. As a result, fifty-five patients with NDLVC were included in the study, with a mean age of 52 ± 15 years (Table 1). The mean follow-up was 39.5 ± 8.6 months. The majority were male (80%), and most patients (80%) were classified as New York Heart Association (NYHA) class I at baseline. The remaining participants were NYHA class II (18%) or III (1.8%). Regarding pharmacologic therapy, 78% of patients were receiving beta-blockers, 47% were on sodium–glucose co-transporter 2 inhibitors (SGLT2i), 40% on renin–angiotensin–aldosterone system inhibitors (RAASi), 29% on angiotensin receptor–neprilysin inhibitors (ARNI), and 51% were treated with mineralocorticoid receptor antagonists (MRA).

The median QRS duration was 96 ms (IQR: 85–112 ms). Conduction abnormalities were infrequent, with 11% presenting with LBBB and 3.6% with RBBB.

Cardiac magnetic resonance imaging revealed a mean left ventricular end-diastolic volume index (LVEDVi) of 99 mL/m^2^ and right ventricular end-diastolic volume index (RVEDVi) of 85 ± 16 mL/m^2^. The average left ventricular ejection fraction (LVEF) was 44 ± 10%, while the right ventricular ejection fraction (RVEF) averaged 54 ± 9%. At baseline, 14 patients (25%) had LVEF < 40%, 25 patients (45%) had LVEF between 40–50%, and 16 patients (29%) had preserved LVEF > 50%. LGE was detected in 69% of patients, with the majority of them involving one to three segments (of a seventeen-segment LV model). A small subset (9%) exhibited more extensive enhancement involving ≥5 segments.

### 3.2. Differences in Holter Parameters Between Visits (Figure 1)

Table 2 presents a comparative analysis of key 24 h Holter-derived parameters between two follow-up time points (Visit A and Visit B) a year apart. As earlier described, each one of these ECG-derived indices represents a risk marker for VA and SCD as explored in previous studies.

In terms of ventricular ectopy, the median burden of PVCs over 24 h showed no significant variation between the two time points (488 [IQR: 12–2742] vs. 432 [35–3576] PVCs/24 h, *p* = 0.524). Similarly, the presence of NSVT was relatively consistent, occurring in 29% of patients at baseline and in 35% at follow-up (*p* = 0.579). Notably, the median number of NSVT complexes per patient remained constant in both visits, as did the peak ventricular rate during NSVT episodes (median 120 vs. 116 bpm, *p* = 0.721). Sustained VT (SVT) episodes were rare, occurring in only two patients at Visit A and one patient at Visit B, with no significant change observed (*p* = 1.000).

Measures of ventricular repolarization, including the mean 24 h QTc interval corrected using the Fridericia formula, remained virtually identical between visits (mean 437 ms at both time points, *p* = 0.947).

Similarly, time-domain heart rate variability assessed by SDNN showed a non-significant tendency toward lower values at Visit B (160 ms vs. 149 ms, *p* = 0.284), while DC, a vagal-specific index of autonomic modulation, remained stable (4.8 vs. 5.0 ms, *p* = 0.475). TO did not differ significantly between visits, but a small yet statistically significant decline in TS was noted (median 5 [3–14] vs. 5 [4–9] ms/RRI, *p* = 0.018.

TWA, a marker of repolarization instability, was detected in 31% of patients at Visit A and in 44% at Visit B, though this increase was not significant (*p* = 0.169). Late potentials, assessed via signal-averaged ECG, were also present in a similar proportion of patients at both time points (45% vs. 44%, *p* = 1.000).

To capture temporal fluctuations in arrhythmic substrate and autonomic function markers—despite the absence of significant differences at the group level—we analyzed the proportion of patients who transitioned from a positive to a negative risk marker (or vice versa) between visits (Table 3). Overall, 67.3% of patients experienced at least one categorical change in the variables examined (95% CI: 65.9–68.5%, *p* < 0.001).

When analyzed individually, PVC burden, using a threshold of ≥1000 PVCs/24 h, changed in 23.6% of patients (95% CI: 22.5–25.1%, *p* < 0.001), and a similar transition rate was observed for NSVT, with 23.6% of patients showing either new onset or resolution of NSVT between visits (*p* < 0.001).

In contrast, TWA, showed the highest degree of change, with 34.5% of patients shifting status between visits (95% CI: 33.3–35.9%, *p* < 0.001). Late potentials transitioned in 16.4% of patients (95% CI: 15.4–17.8%, *p* < 0.001).

Table 4 examines whether changes in Holter-derived electrophysiological parameters between Visit A and Visit B are associated with adverse clinical outcomes, specifically HF hospitalization and VA hospitalization.

For HF hospitalization, which occurred in six patients during follow-up, none of the continuous or categorical variables were significantly predictive. For instance, the odds ratio (OR) for an increase in PVC burden as a continuous variable was 1.00 (95% CI: 1.00–1.00, *p* = 0.682), and the binary categorization of PVCs ≥ 1000/24 h vs. <1000/24 h was also an insignificant predictor. Similarly, transitions in NSVT beats, rate, or burden did not demonstrate a significant association with HF hospitalization risk, nor did prolongation of repolarization (QTc), worsening of autonomic function indices (DC, TO, TS), or new emergence of late potentials.

In terms of VA hospitalization, eight out of fifty-five (14.5%) patients experienced sustained VA that required hospitalization, three of whom where ICD shocks (two in the >200 bpm zone and one shock at 173–200 bpm), three self-terminated monomorphic VTs recorded in outpatient Holter monitoring for palpitations, and the rest were sustained, hemodynamically tolerated, monomorphic VTs. The only significant predictor was SDNN (OR: 0.98; 95% CI: 0.97–0.99; *p* = 0.006. The predictive value of SDNN difference for VA hospitalization was consistent for patients with and without LGE (*p*_interaction_ = 0.238). Similar results were noted for TS difference (*p*_interaction_ = 0.294). Regarding the ROC analysis, the best cutoff value (maximal Youden index) for SDNN difference was 0.17, providing a sensitivity of 62.5% and a specificity of 87.2% for VA hospitalization. The respective value for TS difference was 0.12, corresponding to a sensitivity of 83.3% and a specificity of 71% for predicting VA hospitalization.

Other parameters showed numerical trends without reaching statistical significance. For example, an increase in T-wave alternans was associated with a more than two-fold (though non-significant) increase in VA hospitalization risk (OR: 2.22; 95% CI: 0.40–10.75; *p* = 0.326), and the presence of late potentials conferred a non-significant OR of 2.10 (95% CI: 0.10–19.27; *p* = 0.546). Notably, even composite worsening of any risk marker (“any increase”) was not associated with significant risk of either HF (OR: 0.48; *p* = 0.421) or VA hospitalization (OR: 1.89; *p* = 0.417).

## 4. Discussion

This study sought to examine whether close follow-up through serial 24 h Holter, ECG, and SAECG monitoring better categorizes patients who would benefit from primary risk stratification for major cardiac adverse events. In this manner, a comprehensive longitudinal assessment of noninvasive ECG risk markers for SCD was performed for temporal variability over one year and their associations with clinical outcomes, specifically HF and VA hospitalization, were examined.

Our findings demonstrate that most noninvasive ECG markers remained stable over a one-year period, with no significant group-level changes in PVC burden, NSVT, mean QTc, heart rate variability (SDNN), or vagal modulation (TO, DC). The only marker that showed a statistically significant change was TS, which declined modestly between visits. TS reflects baroreflex sensitivity and vagal reactivity, and its deterioration may signal subclinical progression of autonomic dysfunction [13]. Despite its statistical significance, the clinical implications of this isolated change remain uncertain given the absence of parallel shifts in other autonomic indices or corresponding clinical events.

What is of great importance is that, when assessed at the individual level, a striking degree of temporal variability was observed. Nearly 70% of patients experienced a categorical transition (positive to negative or vice versa) in at least one ECG risk marker, with TWA demonstrating the highest individual transition rate (34.5%), followed by NSVT and PVC burden (each 23.6%). This variability demonstrates that a single assessment offers only a “snapshot” of a patient’s arrhythmic status and does not fully reflect their overall condition. To our knowledge, only one other study with a similar rationale has been published, examining patients post-myocardial infarction with an LVEF ≥ 40% [20]. In that cohort, changes in ECG indices at the individual level were observed in 14% of patients over a one-year follow-up. Ischemic cardiomyopathy typically involves a well-characterized and relatively stable arrhythmic substrate, barring the occurrence of new coronary events. In contrast, NDLVC is marked by significant heterogeneity and a still poorly defined arrhythmogenic substrate. This is reflected in our study by the substantially higher rate of change in the same indices between assessments, observed in 67% of patients.

Despite the significant within-subject transitions in several risk markers, most did not translate into meaningful differences in clinical outcomes during the follow-up period. We compared patients whose one or more ECG parameters worsened between the first and second visits to those whose parameters remained stable or improved. HF hospitalization was not associated with any ECG variable at either time point or with their longitudinal changes. Similarly, VA hospitalization was not predicted by categorical shifts or continuous changes in most arrhythmic markers, including PVC burden, NSVT, or TWA. However, a notable exception was SDNN, where a decline over time was significantly associated with increased odds of VA hospitalization. SDNN, a measure of total heart rate variability, has long been recognized as a surrogate for autonomic tone and is consistently associated with adverse outcomes in various cardiac populations [21,22,23]. Its predictive value in this study suggests that dynamic deterioration in autonomic regulation may be a more robust marker of arrhythmic risk than isolated ectopic events or conduction abnormalities.

This study has several clinical implications. First, it reinforces the concept that patients with NDLVC, despite preserved LV dimensions, represent an at-risk population for adverse events and this should not be overlooked. Second, it demonstrates that noninvasive ECG parameters are subject to substantial temporal variability over follow-up, but this does not necessarily correlate with major adverse cardiac events.

## 5. Limitations

This study has several limitations that warrant consideration. First, the relatively small sample size (*n* = 55) may have limited the statistical power to detect associations between temporal changes in ECG markers and clinical outcomes, particularly for less frequent events such as VA hospitalization or SCD. Second, the follow-up period, although averaging over three years, may still be insufficient to capture the full spectrum of cardiac risk. Third, while efforts were made to standardize data acquisition—including centralized Holter analysis and consistent ECG definitions—measurement variability and occasional technical limitations inherent to ambulatory ECG monitoring cannot be entirely excluded. Also, some degree of temporal variability is inherent to this method of ECG recording. Finally, as this was a single-center study conducted in a tertiary care setting, generalizability to broader populations may be limited.

## Figures and Tables

**Figure 1:**
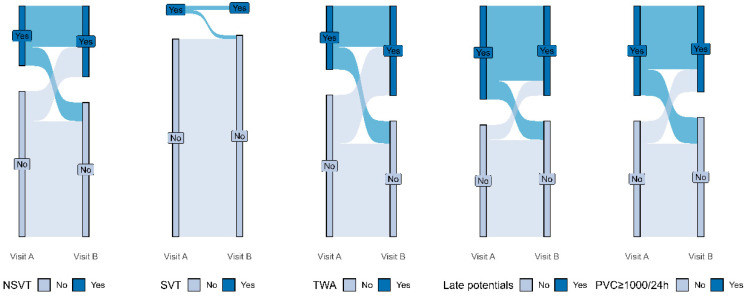
Sankey plots demonstrating transitions in arrhythmic markers between visits. The wider the link, the greater the quantity it represents. Abbreviations: NSVT, non-sustained ventricular tachycardia; SVT, supraventricular tachycardia; TWA, T-wave alternans; PVC; premature ventricular complex.

**Table 1:** Baseline participants’ characteristics.

Variable	Distribution
Age (years)	52 (15)
Sex	
Female	11/55 (20)
Male	44/55 (80)
NYHA class	
NYHA I	44/55 (80)
NYHA II	10/55 (18)
NYHA III	1/55 (1.8)
Beta blockers	43/55 (78)
SGLT2i	26/55 (47)
RAASi	22/55 (40)
ARNI	16/55 (29)
MRA	28/55 (51)
QRS (ms)	96 (85, 112)
RBBB	2/55 (3.6)
LBBB	6/55 (11)
LVEDVi (mL/m^2^)	99 (86, 109)
RVEDVi (mL/m^2^)	85 (16)
RVEF (%)	54 (9)
LVEF (%)	44 (10)
LGE segments (n)	
0	17/55 (31)
1	11/55 (20)
2	10/55 (18)
3	6/55 (11)
4	4/55 (7.3)
5	3/55 (5.5)
6	1/55 (1.8)
9	2/55 (3.6)
17	1/55 (1.8)
LGE presence	38/55 (69)

Abbreviations: NYHA, New York Heart Association; SGLT2, sodium–glucose transport protein 2 inhibitors; RAASi, renin–angiotensin–aldosterone inhibitors; ARNI, angiotensin receptor/neprilysin inhibitor; MRA, mineralocorticoid receptor antagonists; RBBB, right bundle branch block; LBBB, left bundle branch block; LVEDVi, left ventricular end-diastolic volume index; RVEDVi, right ventricular end-diastolic volume index; RVEF, right ventricular ejection fraction; LVEF, left ventricular ejection fraction; LGE, late gadolinium enhancement.

**Table 2:** Differences in Holter parameters between visits.

Variable	Visit A N = 55	Visit B N = 55	*p*-Value
PVCs (n/24 h)	488 (12, 2742)	432 (35, 3576)	0.524
PVC (≥1000/24 h vs. <1000/24 h)	24/55 (44%)	23/55 (42%)	1
NSVT	16/55 (29%)	19/55 (35%)	0.579
NSVT complexes (n)	0.0 (0.0, 3.0)	0.0 (0.0, 4.0)	0.506
NSVT rate (bpm)	0 (0, 120)	0 (0, 116)	0.721
SVT	2/55 (3.6%)	1/55 (1.8%)	1
QTc Fridericia (ms)	437 (28)	437 (30)	0.947
SDNN (ms)	160 (51)	149 (57)	0.284
Turbulence onset (%)	−0.010 (−0.020, 0.000)	−0.010 (−0.025, 0.000)	0.183
Turbulence slope (ms/rri)	5 (3, 14)	5 (4, 9)	**0.018**
DC (ms)	4.8 (2.3, 7.3)	5.0 (2.0, 7.9)	0.475
T-wave alternans	17/55 (31%)	24/55 (44%)	0.169
Late potentials	25/55 (45%)	24/55 (44%)	1

Abbreviations: PVC, premature ventricular complex; NSVT, non-sustained ventricular tachycardia; bpm, beats per minute; ms, milliseconds; SVT, sustained ventricular tachycardia; SDNN, standard deviation of the normal-to-normal beats; DC, deceleration capacity.

**Table 3:** Estimated proportions for overall transitions between visits for each categorical variable.

Variable	Proportion of Transition (95% CI)	*p*-Value
PVCs (≥1000/24 h vs. <1000/24 h)	23.6% (22.5–25.1)	<0.001
NSVT	23.6% (22.5–25.1)	<0.001
SVT	1.8% (1.2–3.2)	<0.001
T-wave alternans	34.5% (33.3–35.9)	<0.001
Late potentials	16.4% (15.4–17.8)	<0.001
Any change	67.3% (65.9–68.5)	<0.001

Abbreviations: CI, confidence interval; PVC, premature ventricular complex; NSVT, non-sustained ventricular tachycardia; SVT, sustained ventricular tachycardia.

**Table 4:** Association between differences in Holter parameters and clinical outcomes.

Holter Parameters (Difference Between Visit A and B)	HF Hospitalization (6/55)		VA Hospitalization (8/55)	
	OR with 95% CI	*p*-Value	OR with 95% CI	*p*-Value
PVCs (n/24 h)	1 (1, 1)	0.682	1 (1, 1)	0.667
PVCs (≥1000/24 h vs. <1000/24 h)	0 (NA, +Inf)	0.995	0 (NA, +Inf)	0.995
NSVT	0 (NA, +Inf)	0.994	0.82 (0.04, 5.71)	0.859
NSVT complexes (n)	0.97 (0.8, 1.04)	0.667	1.04 (0.99, 1.11)	0.142
NSVT rate (bpm)	1 (0.99, 1.01)	0.616	1 (0.99, 1.01)	0.939
QTc Fridericia (ms)	1.01 (0.97, 1.04)	0.728	1.01 (0.98, 1.04)	0.581
SDNN (ms)	1.01 (0.99, 1.02)	0.304	**0.98 (0.97, 0.99)**	**0.006**
Turbulence onset (%)	36.64 (0, +Inf)	0.868	9721.93 (0, +Inf)	0.582
Turbulence slope (ms/rri)	1.06 (0.91, 1.4)	0.62	0.94 (0.86, 1.03)	0.149
DC (ms)	0.99 (0.84, 1.13)	0.939	1 (0.87, 1.13)	0.95
T-wave alternans	0.62 (0.03, 4.35)	0.673	2.22 (0.4, 10.75)	0.326
Late potentials	3.07 (0.14, 29.97)	0.369	2.1 (0.1, 19.27)	0.546
Any increase	0.48 (0.06, 2.7)	0.421	1.89 (0.42, 10.1)	0.417

Abbreviations: HF, heart failure; VA, ventricular arrhythmia; OR, odds ratio; CI, confidence interval; ND, not defined; Inf, infinite; PVC, premature ventricular complex; NSVT, non-sustained ventricular tachycardia; bpm, beats per minute; ms, milliseconds; SVT, sustained ventricular tachycardia; SDNN, standard deviation of the normal-to-normal beats; DC, deceleration capacity.

## Data Availability

The data presented in this study are available on request from the corresponding author. The data are not publicly available due to privacy and ethical restrictions.

## References

[1] Arbelo E., Protonotarios A., Gimeno J.R., Arbustini E., Barriales-Villa R., Basso C., Bezzina C.R., Biagini E., Blom N.A., de Boer R.A. (2023). 2023 ESC Guidelines for the management of cardiomyopathies. Eur. Heart J..

[2] Gueli I.A., Aimo A., Alderotti B., Trimarchi G., Bellisario I., Todiere G., Grigoratos C., De Gori C., Clemente A., Fabiani I. (2025). Arrhythmic risk prediction in non-dilated left ventricular cardiomyopathy: The role of overlap with arrhythmogenic cardiomyopathy. Int. J. Cardiol..

[3] Pinto Y.M., Elliott P.M., Arbustini E., Adler Y., Anastasakis A., Böhm M., Duboc D., Gimeno J., de Groote P., Imazio M. (2016). Proposal for a revised definition of dilated cardiomyopathy, hypokinetic non-dilated cardiomyopathy, and its implications for clinical practice: A position statement of the ESC working group on myocardial and pericardial diseases. Eur. Heart J..

[4] Cuschieri S. (2019). The STROBE guidelines. Saudi J. Anaesth..

[5] Kawel-Boehm N., Hetzel S.J., Ambale-Venkatesh B., Captur G., Francois C.J., Jerosch-Herold M., Salerno M., Teague S.D., Valsangiacomo-Buechel E., van der Geest R.J. (2020). Reference ranges (“normal values”) for cardiovascular magnetic resonance (CMR) in adults and children: 2020 update. J. Cardiovasc. Magn. Reson..

[6] Crawford M.H., Bernstein S.J., Deedwania P.C., DiMarco J.P., Ferrick K.J., Garson A., Smith S.C. (1999). Guidelines for ambulatory electrocardiography. A report of the American College of Cardiology/American Heart Association Task Force on Practice Guidelines (Committee to Revise the Guidelines for Ambulatory Electrocardiography). Developed in collaboration with the North American Society for Pacing and Electrophysiology. J. Am. Coll. Cardiol..

[7] Gatzoulis K.A., Carlson M.D., Biblo L.A., Rizos I., Gialfos J., Toutouzas P., Waldo A.L. (1995). Time domain analysis of the signal averaged electrocardiogram in patients with a conduction defect or a bundle branch block. Eur. Heart J..

[8] Castrichini M., De Luca A., De Angelis G., Neves R., Paldino A., Dal Ferro M., Barbati G., Medo K., Barison A., Grigoratos C. (2024). Magnetic Resonance Imaging Characterization and Clinical Outcomes of Dilated and Arrhythmogenic Left Ventricular Cardiomyopathies. J. Am. Coll. Cardiol..

[9] Milaras N., Dourvas P., Doundoulakis I., Sotiriou Z., Nevras V., Xintarakou A., Laina A., Soulaidopoulos S., Zachos P., Kordalis A. (2023). Noninvasive electrocardiographic risk factors for sudden cardiac death in dilated ca rdiomyopathy: Is ambulatory electrocardiography still relevant?. Heart Fail. Rev..

[10] Grimm W., Christ M., Maisch B. (2005). Long runs of non-sustained ventricular tachycardia on 24-hour ambulatory electrocardiogram predict major arrhythmic events in patients with idiopathic dilated cardiomyopathy. Pacing Clin. Electrophysiol..

[11] Kadish A., Dyer A., Daubert J.P., Quigg R., Estes N.M., Anderson K.P., Calkins H., Hoch D., Goldberger J., Shalaby A. (2004). Prophylactic defibrillator implantation in patients with nonischemic dilated cardiomyopathy. N. Engl. J. Med..

[12] Grimm W., Christ M., Bach J., Müller H.H., Maisch B. (2003). Noninvasive arrhythmia risk stratification in idiopathic dilated cardiomyopathy: Results of the Marburg Cardiomyopathy Study. Circulation.

[13] Klingenheben T., Ptaszynski P., Hohnloser S.H. (2008). Heart rate turbulence and other autonomic risk markers for arrhythmia risk stratification in dilated cardiomyopathy. J. Electrocardiol..

[14] Demming T., Sandrock S., Kuhn C., Kotzott L., Tahmaz N., Bonnemeier H. (2016). Deceleration capacity: A novel predictor for total mortality in patients with non-ischemic dilated cardiomyopathy. Int. J. Cardiol..

[15] Yang Y., Wang F., Zou C., Dong H., Huang X., Zhou B., Li X., Yang X. (2018). Male Patients with Dilated Cardiomyopathy Exhibiting a Higher Heart Rate Acceleration Capacity or a Lower Deceleration Capacity Are at Higher Risk of Cardiac Death. Front. Physiol..

[16] Arsenos P., Gatzoulis K.A., Dilaveris P., Gialernios T., Sideris S., Lazaros G., Stefanadis C. (2011). The rate-corrected QT interval calculated from 24-hour Holter recordings may serve as a significant arrhythmia risk stratifier in heart failure patients. Int. J. Cardiol..

[17] Verrier R.L., Klingenheben T., Malik M., El-Sherif N., Exner D.V., Hohnloser S.H., Rosenbaum D.S. (2011). Microvolt T-wave alternans physiological basis, methods of measurement, and clinical utility–consensus guideline by International Society for Holter and Noninvasive Electrocardiology. J. Am. Coll. Cardiol..

[18] Abraham W.T., Psotka M.A., Fiuzat M., Filippatos G., Lindenfeld J., Mehran R., Ambardekar A.V., Carson P.E., Jacob R., Januzzi J.L. (2020). Standardized definitions for evaluation of heart failure therapies: Scientific expert panel from the Heart Failure Collaboratory and Academic Research Consortium. Eur. J. Heart Fail..

[19] Kumar A., Avishay D.M., Jones C.R., Shaikh J.D., Kaur R., Aljadah M., Kichloo A., Shiwalkar N., Keshavamurthy S. (2021). Sudden cardiac death: Epidemiology, pathogenesis and management. Rev. Cardiovasc. Med..

[20] Xenogiannis I., Gatzoulis K.A., Flevari P., Ikonomidis I., Iliodromitis E., Trachanas K., Vlachos K., Arsenos P., Tsiachris D., Tousoulis D. (2020). Temporal changes of noninvasive electrocardiographic risk factors for sudden cardiac death in post-myocardial infarction patients with preserved ejection fraction: Insights from the PRESERVE-EF study. Ann. Noninvasive Electrocardiol..

[21] Karcz M., Chojnowska L., Zareba W., Ruzyłło W. (2003). Prognostic significance of heart rate variability in dilated cardiomyopathy. Int. J. Cardiol..

[22] Milaras N., Kordalis A., Tsiachris D., Sakalidis A., Ntalakouras I., Pamporis K., Dourvas P., Apostolos A., Sotiriou Z., Arsenos P. (2024). Ischemia testing and revascularization in patients with monomorphic ventricular tachycardia: A relic of the past?. Curr. Probl. Cardiol..

[23] Pamporis K., Karakasis P., Sagris M., Zarifis I., Bougioukas K.I., Pagkalidou E., Milaras N., Samaras A., Theofilis P., Fragakis N. (2024). Mineralocorticoid receptor antagonists in heart failure with reduced ejection fraction: A systematic review and network meta-analysis of 32 randomized trials. Curr. Probl. Cardiol..

